# Characterisation of fibroblast-like synoviocytes from a murine model of joint inflammation

**DOI:** 10.1186/ar4158

**Published:** 2013-01-29

**Authors:** Rowan S Hardy, Claudia Hülso, Yingling Liu, Sylvia J Gasparini, Colette Fong-Yee, Jinwen Tu, Shihani Stoner, Paul M Stewart, Karim Raza, Mark S Cooper, Markus J Seibel, Hong Zhou

**Affiliations:** 1 Bone Research Program, ANZAC Research Institute, University of Sydney, Hospital Road, Concord, Sydney, 2139, Australia; 2 Centre for Endocrinology, Diabetes and Metabolism, Institute of Biomedical Research, University of Birmingham, Vincent Drive, Birmingham, B15 2TT, UK; 3 Department of Immunity and Infection, Institute of Biomedical Research, University of Birmingham, Vincent Drive, Birmingham, B15 2TT, UK; 4 Department of Endocrinology & Metabolism, Concord Repatriation Hospital, University of Sydney, Hospital Road, Concord, Sydney, 2139, Australia

## Abstract

**Introduction:**

Fibroblast-like synoviocytes (FLS) play a central role in defining the stromal environment in inflammatory joint diseases. Despite a growing use of FLS isolated from murine inflammatory models, a detailed characterisation of these cells has not been performed.

**Methods:**

In this study, FLS were isolated from inflamed joints of mice expressing both the T cell receptor transgene KRN and the MHC class II molecule Ag7 (K/BxN mice) and their purity in culture determined by immunofluorescence and real-time reverse transcription polymerase chain reaction (real-time RT-PCR). Basal expression of proinflammatory genes was determined by real-time RT-PCR. Secreted interleukin 6 (IL-6) was measured by enzyme-linked immunosorbent assay (ELISA), and its regulation by tumor necrosis factor-alpha (TNF-α and corticosterone (the major glucocorticoid in rodents) measured relative to other mesenchymal cell populations.

**Results:**

Purity of FLS culture was identified by positive expression of fibronectin, prolyl 4-hydroxylase, cluster of differentiation 90.2 (CD90.2) and 248 (CD248) in greater than 98% of the population. Cultured FLS were able to migrate and invade through matrigel, a process enhanced in the presence of TNF-α. FLS isolated from K/BxN mice possessed significantly greater basal expression of the inflammatory markers IL-6, chemokine ligand 2 (CCL-2) and vascular cell adhesion molecule 1 (VCAM-1) when compared to FLS isolated from non-inflamed tissue (IL-6, 3.6 fold; CCL-2, 11.2 fold; VCAM-1, 9 fold; *P *< 0.05). This elevated expression was abrogated in the presence of corticosterone at 100 nmol/l. TNF-α significantly increased expression of all inflammatory markers to a much greater degree in K/BxN FLS relative to other mesenchymal cell lines (K/BxN; IL-6, 40.8 fold; CCL-2, 1343.2 fold; VCAM-1, 17.8 fold; ICAM-1, 13.8 fold; *P *< 0.05), with secreted IL-6 mirroring these results (K/BxN; con, 169 ± 29.7 versus TNF-α, 923 ± 378.8 pg/ml/1 × 10^5 ^cells; *P *< 0.05). Dose response experiments confirmed effective concentrations between 10 and 100 nmol/l for corticosterone and 1 and 10 ng/ml for TNF-α, whilst inflammatory gene expression in FLS was shown to be stable between passages four and seven.

**Conclusions:**

This study has established a well characterised set of key inflammatory genes for *in vitro *FLS culture, isolated from K/BxN mice and non-inflamed wild-type controls. Their response to both pro- and anti-inflammatory signalling has been assessed and shown to strongly resemble that which is seen in human FLS culture. Additionally, this study provides guidelines for the effective characterisation, duration and treatment of murine FLS culture.

## Introduction

Rheumatoid arthritis (RA) is a chronic autoimmune inflammatory disease that leads to progressive damage to articular and periarticular structures. It is characterised by hyperplasia of fibroblast-like synoviocytes (FLS) within the synovium and recruitment of multiple leukocyte populations that drive the inflammatory process [1]. Our understanding of the aetiology and pathology of inflammatory joint disease has been greatly advanced through the use of animal models [2-7]. Despite variation in the targeting and severity of inflammation, these models all result in some degree of damage to articular cartilage or surrounding bone. Importantly, they all present with synovial hyperplasia, characterised by hyperproliferation of FLS. Consequently, these models have been important in investigating the roles of FLS in the mediation of inflammatory bone loss [8-11].

FLS are stromal cells of mesenchymal origin that demonstrate highly active behaviour, producing a range of extracellular matrix components and secreted factors that help maintain the normal environment of the synovial fluid and articular surface [12-14]. In addition, these cells have been shown to be key mediators in the maintenance of inflammation and in driving joint destruction during synovitis. During joint inflammation, FLS take on an aggressive, invasive phenotype, breaking down cartilage by the action of matrix metalloproteinases, whilst their production of secreted factors such as receptor activator of nuclear factor kappa-B (NFκ-B) ligand (RANKL) promotes osteoclast differentiation, survival and activity, contributing to bone erosion and juxta-articular osteoporosis [15,16].

The expression of multiple proinflammatory adhesion markers and cytokines is upregulated in human FLS isolated from inflamed RA joints, which impact on leukocyte migration, survival and activation [17-20]. Amongst the cytokines secreted by FLS, IL-6 has been shown to play a significant role in the pathology of RA, and is strongly upregulated during inflammation, having diverse roles both locally and systemically [21,22]. This activated inflammatory phenotype observed in human FLS has been shown to be maintained over prolonged cell culture, even in the absence of proinflammatory cytokines [23-27]. Consequently, FLS are targets for disease-modifying and anti-inflammatory drugs, which has resulted in a substantial body of work characterising these cells and examining their contribution in human disease using well-defined methods of isolation [28]. In contrast, isolation of FLS from murine models of inflammation has proved more challenging as a result of the smaller size of affected tissues. Consequently, both murine FLS isolation methods and the resulting cell cultures have yet to be as well characterised as their human counterparts. This lack of knowledge hinders research utilising these cells *in vitro*, used to delineate the pathology in mouse models of inflammatory joint disease.

The aims of this study were to comprehensively characterise the inflammatory phenotype of FLS isolated and cultured from the K/BxN model of inflammatory joint disease. In particular, we were interested in investigating the expression of inflammatory markers relative to normal mesenchymal cell populations, and determining how this inflammatory phenotype is maintained over prolonged cell culture.

## Materials and methods

### Mouse models

K/BxN mice that spontaneously develop arthritis were generated as previously described by Kouskoff *et al. *[29,30]. Male KRN transgenic mice (kindly provided by Le Centre Européen de Recherche en Biologie et en Médecine) were crossed with female NOD mice. Resulting K/BxN mice exhibit significant reproducible joint inflammation at 60 days. Animals were kept at the animal facility of the ANZAC Research Institute, in accordance with Institutional Animal Welfare Guidelines and according to an approved protocol. Ethical approval was given by the Sydney Local Health District Animal Welfare Committee under protocols No 2008/043 and 2012/006. Clinical scores of joint swelling were determined using the method described by both Buttgereit and Lee *et al. *[31,32]. Scores of joint swelling are displayed in Table 1. To generate one FLS cell line, tissues from all limbs, from a minimum of three mice from the same litter were combined. Wild-type (WT) mice from the same background were used to generate FLS from non-inflamed joints.

**Table 1:** K/BxN clinical scores.

	Average clinical score (*n *= 3 per litter)					
	**Weight**	**Wrist (L)**	**Wrist (R)**	**Ankle (L)**	**Ankle (R)**	**Total**
K/BxN litter 1	27.43 + 2.2	3.00	2.83	2.83	2.67	11.33 ± 0.6
K/BxN litter 2	23.26 + 3.5	2.67	3.00	2.67	3.00	11.33 ± 0.3
K/BxN litter 3	28.36 + 2.6	2.67	3.00	3.00	3.00	11.66 ± 0.4
WT litter 1	24.31 + 1.1	0.00	0.00	0.00	0.00	0 ± 0
WT litter 2	25.01 + 2.9	0.00	0.00	0.00	0.00	0 ± 0

### FLS isolation and culture

The method used for the isolation of FLS from synovial tissue was modified from a method previously described [33]. FLS lines one, two and three were isolated from K/BxN litters one to three respectively. Mice were euthanized by cervical dislocation prior to dissection of inflamed joints. The front and hind limbs were separated at the humerus/ulna/radius and femur/fibula/tibia junctions, respectively. Limbs were washed in DMEM, high glucose, GlutaMAX (Life Technologies, Grand Island, NY, USA) supplemented with 10% heat-inactivated FBS, 100 U/ml penicillin and 100 mg/ml streptomycin. All further dissection was performed with tissues immersed in culture media. Attached skin, nail, muscle and tendon were removed by microdissection taking care to avoid damaging the bones. Any damaged bones were immediately removed from the cell isolation process to prevent contamination of FLS culture by cells from the bone marrow compartment. The individual bones of the paws were then isolated by dissection to open up the joint spaces and expose the synovial tissues. Dissected bones with synovial tissue were incubated in 20 ml of culture media containing 1 mg/ml of collagenase type 4 (Worthington Biochemical Corp., Freehold, NJ, USA, 220 U/mg) and 0.1 mg/ml of deoxyribonuclease I (Sigma-Aldrich, Castle Hill, NSW, Australia), shaking vigorously for 1 hr at 37°C. Tissues were vortexed at high speed and the media removed to a fresh tube. Tissues were then resuspended in 20 ml of fresh media, vortexed once again and combined with the former, leaving the tissue and bones behind. The combined media was centrifuged at 1200 rpm for 3 min and the cell pellet resuspended in 20 ml of fresh media and cultured at 37°C, 5% C0_2_. Culture media was changed every three days and cells subcultured at 80 to 90% confluence prior to characterisation at passage four.

### Cell culture

Isolated FLS and the murine mesenchymal C2C12 cell line (ATCC, Manassas, VA, USA) were grown in DMEM high glucose, GlutaMAX (Life Technologies, Grand Island, NY, USA) supplemented with 10% heat-inactivated FBS, 100 U/ml penicillin and 100 mg/ml streptomycin. The partially differentiated murine preosteoblastic cell line, MC3T3-E1 cells (ATCC, USA) were cultured in MEM alpha (Life Technologies, Grand Island, NY, USA) supplemented with 10% heat-inactivated FBS, 100 U/ml penicillin, 100 g/ml streptomycin and 1% L-glutamine. All cells were cultured at 37°C, 5% C0_2 _and the media changed every three days. Cells were sub-cultured at 80 to 90% confluence. Unless stated otherwise, fibroblasts were treated with 10 ng/ml tumour necrosis factor (TNF)-α, (R&D Systems, Abingdon, UK) or 100 nmol/l corticosterone (Sigma-Aldrich, Castle Hill, NSW, Australia) for 24 hrs before harvesting.

### Immunofluorescence

Fluorescence immunohistochemistry was performed on FLS at passage 4 in cell culture. Cells were fixed in 2% paraformaldehyde before incubation with primary antibody for one hr at room temperature. Cells were stained using polyclonal antisera to the following: fibronectin 1:400 (F3648, Sigma-Aldrich, Castle Hill, NSW, Australia), CD248 1:200 (PAB13304, Abnova, Taipei City, Taiwan), CD90.2 1:100 (550543; BD Biosciences Pharminogen, Franklin Lakes, NJ, USA), CD31 1:50 (ab28364, Abcam, Cambridge, UK), and CD68 1:100 (H-255, Santa Cruz Biotechnology, Inc., Santa Cruz, CA, USA). The following secondary antibodies were used for 1 hr at room temperature: goat anti-rabbit immunoglobulin G (IgG) labelled with FITC 1:400 (sc2012, Santa Cruz Biotechnology, Inc., Santa Cruz, CA, USA) and rat anti-mouse IgG labelled with Alexa Fluor 633 1:400 (A21094, Invitrogen, Grand Island, NY, USA). Nuclei were counterstained using TO-PRO™-3 1:400 (T3605, Life Technologies, Melbourne, VIC, Australia) for 1 hr at room temperature. Immunofluorescence was performed using an Olympus (Olympus Corp., Tokyo, Japan) FV5-PSU confocal microscope and analyzed using FLUOVIEW software.

### Matrigel invasion assay

Invasion of FLS was examined across matrigel-coated transwell inserts. Eight micron pore size matrigel-coated inserts (BD Biosciences, San Jose, CA, USA) were incubated in normal FLS culture media for 2 hrs in 24-well plates. 3000 FLS were then seeded in the inner compartment of the transwell system in 0.5 ml of FLS culture media containing 0.1% BSA. 0.75 ml of FLS culture media containing 10% FBS and 50 ng/ml platelet-derived growth factor (PDGF) with or without 10 ng/ml IL-1 or TNF-α stimulant (R&D systems, Abingdon, UK) was then added to the outer compartment. FLS were incubated at 37°C, 5% C0_2 _for 24 hrs. The insert membrane was then isolated and fixed in 10% buffered formalin before washing in PBS and staining with Harris haematoxylin. FLS that invaded through the matrigel and passed through the 8 micron pores were counted.

### RNA extraction/reverse transcription

RNA isolation was performed using the InnuPREP mini kit (Analytik Jena AG, Jena, Germany) following the manufacturer's protocol. First-strand cDNA was synthesized from 1 μg of total RNA by incubating for 1 h at 50°C with SuperScript III reverse transcriptase (Invitrogen, Mulgrave, VIC, Australia) following oligo(dT) priming.

### Real-time PCR

Expression of mRNA for IL-6, chemokine ligand 2 (CCL-2), vascular cell adhesion molecule 1 (VCAM-1), intercellular adhesion molecule 1 (ICAM-1) and bone gamma-carboxyglutamic acid-containing protein (BGLAP) was assessed using IQ SYBR Green Supermix (Bio-Rad Laboratories, Regents Park, NSW, Australia) according to the manufacturer's instructions, using a Bio-Rad iCycler iQ5 real-time PCR detection system. Primers for 18S were used for cDNA normalization. Reactions occurred as follows: 95°C for 2 minutes, 40 cycles of 95°C for 10 seconds, 60°C for 15 seconds and 72°C for 30 seconds. Data were obtained as Ct values (the cycle number at which logarithmic PCR plots cross a calculated threshold line), and used to determine ΔCt values (Ct of target gene - Ct of housekeeping gene) as raw data for gene expression. The fold change in gene expression was determined by subtracting ΔCt values for treated cells from their respective control samples. The resulting ΔΔCt values were then used to calculate fold change in gene expression according to the expression 2^ΔΔCt^. Primer sequences used are summarized in Table 2.

**Table 2:** Primer sequences.

Gene	Fwd	Rvs
18S	CATGATTAAGAGGGACGGC	TTCAGCTTTGCAACCATACTC
IL-6	AGTTGCCTTCTTGGGACTGA	GGTAGCATCCATCATTTCTTTGTA
CCL-2	AGAAGTCATAGCCACTCTCAAGG	TGAACTCTCAGACAGCGAGG
VCAM-1	GGAGACCTGTCACTGTCAACTG	TCCATTTCACCACTGTGTAACC
ICAM-1	AGACACAAGCAAGAAGACCACA	TGACCAGTAGAGAAACCCTCG
BGLAP	GCTCTGTCTCTCTGACCTCACA	TAGATGCGTTTGTAGGCGG
CD68	GCTTCTGCTGTGGAAATGC	GGTAGGTTGATTGTCGTCTGC
P4htm	GATTGTGGAGTTCAGTGAGCC	TTCATCATAGGTCCTGTTGTCTG
CD248	GCCAGCAGATGTGTGTCAA	GTAGGTGCCAGCCATAGGAT

### Immunohistochemistry

Immunohistochemical assessment of CD248 and fibronectin expression within littermates of non-inflamed control and inflamed K/BxN ankle joints were performed. Briefly, following decalcification in EDTA, paraffin-embedded sections were cut and stained with either the fibronectin 1:400 (F3648, Sigma-Aldrich, Castle Hill, NSW, Australia) or CD248 1:400 (PAB13304, Abnova, Taipei City, Taiwan) primary antibodies. A biotinylated anti-rabbit immunoglobulin (Vectastatin ABC kit, Vector Laboratories, Burlingame, CA, USA) served as the secondary antibody. DAB substrate kit for peroxidase (Vector Laboratories, Burlingame, CA, USA) was used for chromagen development. Samples were counterstained with Gill's haematoxylin.

### IL-6 ELISA

IL-6 levels in supernatants from cultured cells were measured using a commercially available sandwich ELISA in accordance with the manufacturer's instructions (R&D systems, Abingdon, UK). Data were expressed as pg IL-6/1 × 10^5 ^cells.

### Statistical analysis

Data are reported as mean ± standard error (SE) of replicate mean values for separate mouse litter cell cultures. One-way ANOVA analysis of variance was performed using SPSS Data Editor (SPSS Inc., Santa Clara, CA, USA).

## Results

### Characterisation of FLS culture

**Figure 1:**
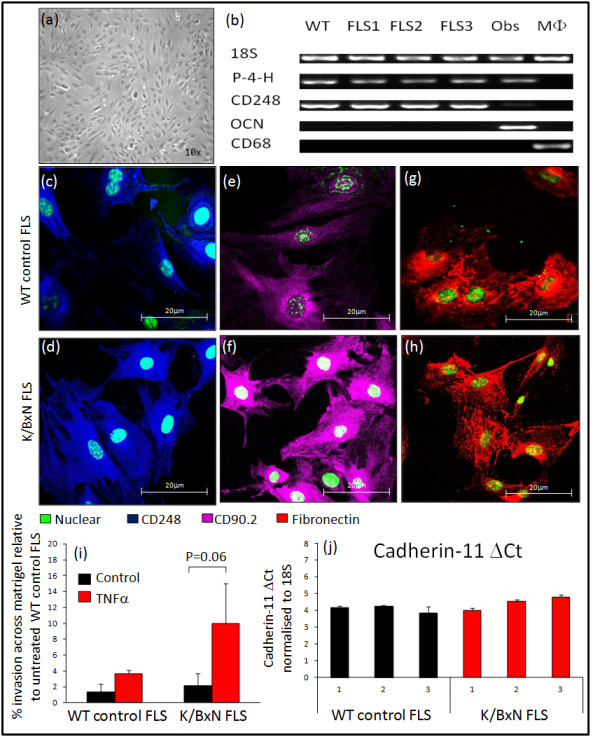
**Validation of FLS culture**. **(a) **Confluent monolayer of K/BxN (fibroblast-like synoviocytes) FLS as observed *in vitro *at 10× magnification. **(b) **mRNA expression of 18S, P4H, CD248, osteocalcin (OCN) and CD68 determined by standard RT-PCR at 35 cycles in one non-inflamed control FLS wild-type (WT), three K/BxN FLS lines (FLS 1 to 3), primary calvarial osteoblasts (OBs) and the macrophage cell line RAW 264.7 (MΦ). Expression of CD248 (blue), CD90.2 (magenta) and fibronectin (red) in non-inflamed FLS **(c, e, g) **and K/BxN FLS **(d, f, h)**, determined by confocal fluorescence immunohistochemistry. **(i) **Invasion of FLS across matrigel-coated transwell inserts relative to untreated non-inflamed control FLS in the presence or absence of TNF-α (ng/ml). **(j) **ΔCt mRNA expression of cadherin-11 normalised for the housekeeping gene 18s. Data presented are from three individual WT control FLS and three K/BxN FLS after loading 25 ng of mRNA for RT-PCR. For Figure 1 c-h, results shown are representative of three separate K/BxN FLS lines. Figure 1b has been cropped, to allow presentation of multiple gels.

For the purpose of characterisation, only cells that survived in culture and actively proliferated beyond passage four were utilised. K/BxN FLS exhibited a classic spindle-shaped fibroblastic phenotype that formed parallel clusters when confluent (Figure 1a). Analysis of cells by quantitative PCR identified significant expression of mRNA for the enzyme prolyl 4-hydroxylase (an enzyme required for fibroblastic collagen synthesis that has previously been shown to be an effective FLS marker [34]) and the synovial fibroblast surface marker CD248 in the three K/BxN FLS lines used in this study (Figure 1b, Additional file 1). In contrast, mRNA expressions of the osteoblast product osteocalcin and macrophage marker CD68 were entirely absent. When analysed by immunohistochemistry, > 98% of FLS stained positively for the stromal mesenchymal marker fibronectin and the synovial fibroblastic surface markers CD90.2 and CD248 (Figure 1c-h). In contrast, less than 2% of cells stained positively for the endothelial marker CD31 or the macrophage marker CD68 (Additional file 2). Examination of invasive behaviour in FLS revealed that both non-inflamed control FLS and K/BxN FLS migrated through the matrigel-coated insert (Figure 1i). Pretreatment with TNF-α for 24 hr prior to seeding resulted in a strong trend towards increasing K/BxN FLS invasion across the matrigel insert (con, 2.16% ± 0.5 vs. TNF-α, 10% ± 4.9 invasion relative to non-inflamed control; *P *= 0.06). PCR analysis of cadherin-11 confirmed positive mRNA expression within 22 cycles in both WT control and K/BxN FLS with no significant differences between groups when normalised to the housekeeping gene 18S (WT con FLS, 4.08 ± 0.24; K/BxN FLS, 4.38 ± 0.34 ΔCt; NS) (Figure 1j). No differences in cadherin-11 mRNA expression were observed following preincubation with TNF-α for 24 hr (Additional file 3).

### CD248 and fibronectin expression *in vivo*

**Figure 2:**
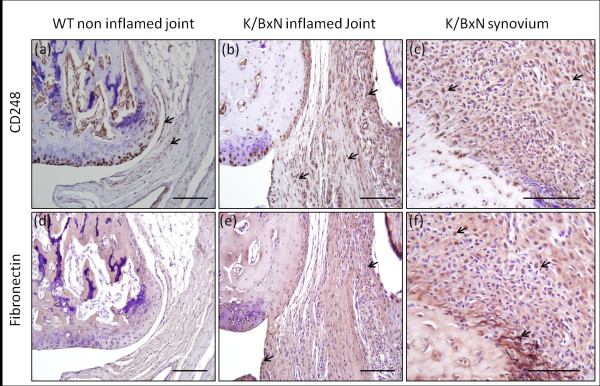
**Synovial CD248 and fibronectin expression *in vivo***. Decalcified paraffin-embedded joint sections were stained for CD248 and fibronectin and counterstained with Gill's haematoxylin. Staining was examined in non-inflamed control **(a, d) **and inflamed K/BxN **(b, e) **joints. **(c, f) **demonstrates the inflamed synovium of K/BxN joints at increased magnification. Black arrows denote positive staining of CD248 and fibronectin respectively. Bars = 100 μm.

To better qualify the use of CD248 and fibronectin as markers for the identification of FLS, we assessed the tissue localization by immunohistochemical stains in both non-inflamed control and inflamed K/BxN joints. Synovial staining for CD248 was relatively weak within the joints of non-inflamed control mice with few positively staining cells (Figure 2a). By contrast, CD248 staining throughout the synovium of inflamed K/BxN joints was markedly elevated compared to non-inflamed controls (Figure 2b, c). Similarly, fibronectin-positive stromal cells were largely absent in non-inflamed control joints, however, their expression was elevated within the synovium of K/BxN inflamed joints (Figure 2d, e, f).

### Fibroblast inflammatory gene expression

**Figure 3:**
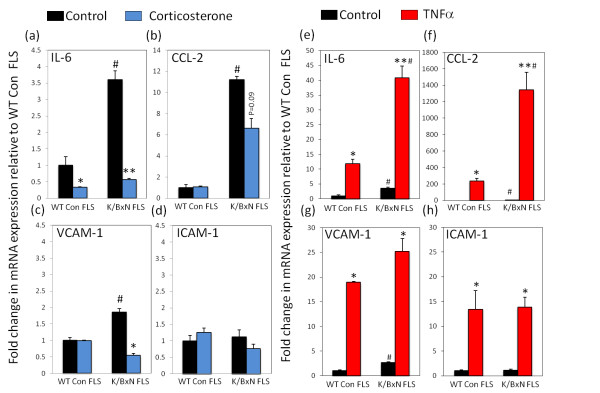
**Inflammatory gene regulation by corticosteone and TNF-α in FLS**. Fold change in mRNA expression of inflammatory genes in (fibroblast-like synoviocytes) FLS, determined by real-time RT-PCR. Expression of mRNA was measured at 16 hr for IL-6, chemokine ligand 2 (CCL-2), vascular cell adhesion molecule 1 (VCAM-1) and intercellular adhesion molecule 1 (ICAM-1) following pretreatment with either corticosterone **(a-d) **(100 nmol/l) or TNFα **(e-h) **(10 ng/ml) for 24 hr. Data were normalized for levels of the housekeeping gene 18S rRNA and presented as fold change in expression (± standard error) relative to untreated wild-type (WT) control FLS. *P < 0.05, **P < 0.001 versus respective untreated control; #*P *> 0.05 versus untreated WT control FLS. Results shown are the combined duplicates of three separate FLS lines and two WT control FLS lines.

We examined the mRNA expression of the proinflammatory cytokine IL-6, the chemokine CCL-2 and the surface markers VCAM-1 and ICAM-1, to obtain a basic measure of the inflammatory profile of FLS isolated from K/BxN mice. Results shown are given as fold change relative to FLS isolated from normal joints in control wild-type mice (WT control FLS) to better assess basal activation of inflammatory markers. Basal expression of IL-6, CCL-2 and VCAM-1 were significantly higher in K/BxN FLS relative to WT control FLS (IL-6, 3.6 ± 0.25 fold; CCL-2, 11.2 ± 0.28 fold; VCAM-1, 9 ± 0.1 fold relative to untreated WT control FLS; *P *< 0.05), whilst no difference was observed with ICAM-1 (Figure 3a, b, c, d). Treatment with the anti-inflammatory glucocorticoid, corticosterone, resulted in a significant decrease in both IL-6 and VCAM-1 expression relative to their respective untreated controls (IL-6, 5.3 ± 0.04 fold; VCAM-1, 3.3 ± 0.07 fold; *P *< 0.05) and led to a strong trend towards decreased CCL-2 expression (1.9 ± 0.9 fold; *P *= 0.09) (Figure 3a, b, c, d). These decreases in IL-6, VCAM-1 and CCL-2 in K/BxN FLS resulted in their expression being comparable to that observed in untreated WT control FLS.

All inflammatory markers were significantly increased in both WT control FLS and K/BxN FLS in response to the proinflammatory cytokine TNF-α (IL-6, 40.8 ± 2.8 fold; CCL-2, 1343.2 ± 362.2 fold; VCAM-1, 25.3 ± 2.6 fold; ICAM-1, 13.8 ± 2.4 fold in K/BxN FLS relative to untreated WT control FLS; *P *< 0.05) (Figure 3e, f, g, h). The increase seen in IL-6 and CCL-2 was significantly greater in K/BxN FLS relative to the increase in WT control FLS (IL-6, 3.45 ± 1.3 fold; CCL-2, 5.7 ± 1.4 fold versus TNF-α induction in WT control FLS; *P *< 0.05).

### Regulation of IL-6 expression in FLS

**Figure 4:**
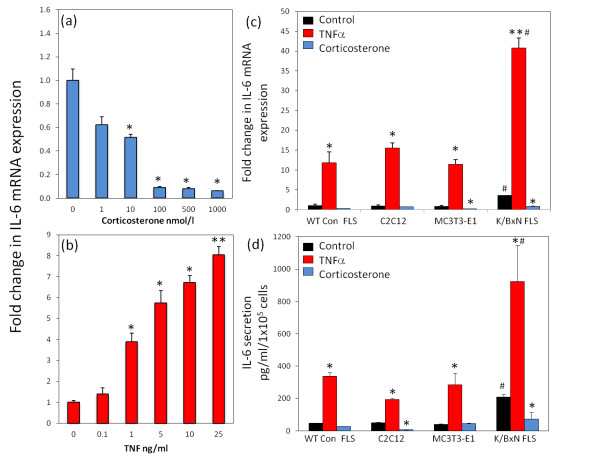
**Regulation of IL-6 in FLS**. Dose response analysis of IL-6 mRNA expression, determined by RT real-time PCR in (fibroblast-like synoviocytes) FLS isolated from K/BxN mice following treatment with **(a) **corticosterone (0, 1, 10, 100, 500, 1000 nmol/l) or **(b) **TNFα (0, 0.1, 1, 5, 10, 25 ng/ml). **(c) **Fold change in IL-6 mRNA expression in wild-type (WT) control FLS, C2C12, MC3T3-E1 and K/BxN FLS, determined by real-time RT-PCR. All mRNA data were normalized for levels of the housekeeping gene 18S rRNA and presented as fold change in expression (± standard error) relative to either untreated control or untreated WT con FLS. **(d) **IL-6 secretion into culture media (pg/ml/100000 cells, ± standard error) in WT con FLS, C2C12, MC3T3-E1 and K/BxN FLS, determined by specific ELISA. Both mRNA and conditioned media were collected at 16 hr following treatment with either control, TNFα (10 ng/ml) or corticosterone (100 nmol/l). *P < 0.05, **P < 0.001 versus respective untreated controls; #*P *< 0.05 versus untreated WT control FLS. Dose response experiments are the combined duplicates of two K/BxN FLS. All other data are the combined duplicates of three separate FLS lines, two WT control FLS lines, two C2C12 repeats and two MC3T3-E1 repeats.

IL-6 mRNA expression was examined in response to the proinflammatory cytokine, TNF-α and the anti-inflammatory glucocorticoid, corticosterone. Time-course analysis of two FLS lines revealed maximal responses between 8 and 24 hr for TNF-α (10 ng/ml) and 8 and 16 hr for corticosterone (100 nmol/l) (Additional file 3). Consequently for all experiments in this study treatments were fixed at 16 hr. Dose response experiments were performed in two FLS lines (Figure 4a, b). TNF-α resulted in a significant increase in IL-6 mRNA expression at 1 ng/ml (3.9 ± 0.41 fold; *P *< 0.05). IL-6 mRNA expression continued to increase in a dose-dependant manner up to the maximum supraphysiological dose used in the study at 25 ng/ml (8 ± 0.23 fold; *P *< 0.001). Treatment with corticosterone resulted in a dose-dependant decrease in IL-6 mRNA expression that was significant at 10 nmol/l (1.9 ± 0.04 fold; *P *< 0.05) with maximal inhibition at 100 nmol/l (11.1 ± 0.02 fold; *P *< 0.05). For all experiments in this study TNF-α and corticosterone treatments were fixed at 10 ng/ml and 100 nmol/l respectively.

**Figure 5:**
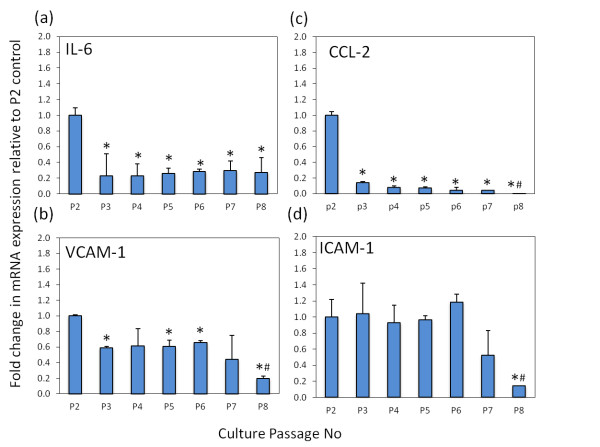
**Regulation of inflammatory markers over prolonged culture**. Fold change in mRNA expression of inflammatory genes in fibroblast-like synoviocytes (FLS), determined by real-time RT-PCR between passages 2 and 8 (P2 to P8). Expression of mRNA was measured at 16 hr for **(a) **IL-6, **(b) **chemokine ligand 2 (CCL-2), **(c) **vascular cell adhesion molecule 1 (VCAM-1) and **(d) **intercellular adhesion molecule 1 (ICAM-1). For each gene product data were normalized for levels of the housekeeping gene 18S rRNA and are presented as fold change in expression (± standard error) relative to the P2 control. *P < 0.05 versus passage 2. #*P *< 0.05 versus passage 3. Results shown are the combined duplicates of two K/BxN FLS.

To examine how the inflammatory phenotype of FLS isolated from inflamed tissues compares to cells isolated from non-inflamed tissues as well as undifferentiated and partially differentiated mesenchymal cell lines, we set up a panel of treatments looking at IL-6 expression in a range of cell types. These included FLS isolated from normal joints in control mice, as well as the undifferentiated mesencyhmal precursor line C2C12 and the partially differentiated osteoblast cell line MC3T3E-1. At the mRNA level, WT control FLS, C2C12 and MC3T3-E1 possessed a similar basal expression of IL-6 (Figure 5c). All lines significantly increased expression of IL-6 in response to the proinflammatory cytokine TNF-α relative to the untreated WT control FLS (WT control FLS, 11.8 ± 2.7 fold; C2C12, 15.6 ± 1.3 fold; MC3T3-E1, 11.4 ± 1.2 fold; *P *< 0.01). Treatment with corticosterone resulted in a significant decrease in IL-6 mRNA expression in the MC3T3-E1 line (5.1 ± 0.05 fold relative to untreated control; *P *< 0.05), whilst no significant decrease was observed in WT control FLS or C2C12. When compared to these cell lines, K/BxN FLS isolated from inflamed tissue had significantly higher basal expression of IL-6 mRNA. Similarly, treatment of K/BxN FLS with TNF-α resulted in a significantly greater upregulation of IL-6 mRNA expression relative to the other cell lines (40.8 ± 2.8 fold increase versus untreated WT control FLS; *P *< 0.001). Treatment with corticosterone significantly reduced expression of IL-6 mRNA in K/BxN FLS relative to untreated control (6.7 ± 0.1 fold; *P *< 0.01). This was comparable to levels observed in WT control FLS.

A very similar pattern was observed for IL-6 secretion into culture media for WT controls FLS, MC3T3-E1 and K/BxN FLS (Figure 5d). No significant difference was observed between basal expression in WT control FLS, C2C12 and MC3T3-E1 (47.6 ± 2.3, 51.3 ± 5.4 and 39.7 ± 9.4 pg/ml/1 × 10^5 ^cells; NS). K/BxN FLS possessed significantly higher basal production of IL-6 relative to WT control FLS, C2C12 and MC3T3-E1 cells (169 ± 29.7 pg/ml/1 × 10^5 ^cells; *P *< 0.05). All lines observed significantly increased IL-6 secretion in response to TNF-α relative to untreated controls (WT control FLS, 338 ± 39.4; C2C12 194 ± 10.2, MC3T3-E1, 285.9 ± 122; K/BxN FLS, 886.9 ± 378.3 pg/ml/1 × 10^5 ^cells; *P *< 0.05). The induction in K/BxN FLS was significantly greater than the induction observed in WT control FLS and MC3T3-E1 cells (*P *< 0.05). Treatment with corticosterone had no significant effect on IL-6 secretion in WT control FLS or MC3T3-E1 cells but resulted in a significant decrease in K/BxN FLS and C2C12 (K/BxN; Con, 169 ± 29.7 versus corticosterone, 79.5 ± 71.3 pg/ml/1 × 10^5 ^cells; *P *< 0.05).

### Inflammatory gene expression with progressive subculture

To assess the maintenance of inflammatory genes over time we collected mRNA in duplicate from one representative FLS culture between passages two and eight (Figure 5a, b, c, d). IL-6, CCL-2, VCAM-1 and ICAM-1 were assessed over these time points. IL-6, CCL-2 and VCAM-1 dropped sharply at passage three compared to passage two in cultures (IL-6, 4.3 ± 0.3 fold; CCL-2, 7.1 ± 0.05 fold; VCAM-1, 1.7 ± 0.02 fold, *P *< 0.05). Expression of IL-6 mRNA remained stable after this point up to passage eight. Similarly, expression of CCL-2, VCAM-1 and ICAM-1 remained stable after passage three up to passage seven with a significant decrease in expression at passage eight relative to passage three (CCL-2, 15.3 ± 0.01 fold; VCAM-1, 3.01 ± 0.03 fold; ICAM-1, 7.1 ± 0.01 fold relative to passages three; *P *< 0.05). Expression of the macrophage marker CD68 also displayed a significant decrease beyond passage three (Additional file 3).

## Discussion

Fibroblast-like synoviocytes have been shown to play a central role in defining the stromal environment in inflammatory joint diseases and to be mediators of joint destruction and persistent inflammation [26,35,36]. To date, extensive research has been performed using human FLS isolated from synovial tissue from patients undergoing joint arthroplasty. These cells have been well characterised in multiple disease states, including RA, osteoarthritis, psoriatic arthritis, juvenile onset arthritis and crystal arthropathy [34,37]. In contrast, despite a growing use of FLS isolated from murine inflammatory models, a similar thorough characterisation of mouse FLS has yet to be performed. In the present study, we successfully isolated FLS from K/BxN mice and characterised multiple inflammatory markers. Cells were characterised on multiple criteria to provide the best confirmation of their origin and purity. These included general stromal fibroblast markers such as fibronectin and prolyl 4-hydroxylase [38] and more specific FLS markers such as CD90.2 [39,40] and CD248 [41]. Alone, CD90.2 stains numerous cell types including thymocyte populations, neurons, epithelial cells and subsets of fibroblasts [42]. By contrast, CD248 has been shown to be a fairly selective marker present in stromal fibroblasts and pericytes within proliferating tissues such as inflammatory lesions, lymphoid tissues, foetal tissues and tumours [43-45]. Of particular interest are reports demonstrating elevated CD248 expression within FLS and pericytes of rheumatoid and psoriatic synovial tissues compared with healthy controls [41]. We observed similar findings within the synovium of inflamed K/BxN joints with greater expression of both CD248 and fibronectin-positive cells compared to non-inflamed controls. The correlation between CD248 and fibronectin within the inflamed synovium and that observed in our *in vitro *cell culture strongly suggests that these cells derive from this tissue. However, it is the combination of CD90.2, CD248 with prolyl 4-hydroxylase and fibronectin that indicate that these cells are truly FLS. Interestingly, we identified expression of CD248 in our non-inflamed control FLS. Although expression of this marker is greatly reduced in the synovium of healthy controls, we were unable to identify significant differences in expression between non-inflamed and inflamed FLS by immunoflourescence. The analysis of CD248 expression in different groups by flow cytometry may provide a better measure of differences in relative expression, as well as allowing us to better distinguish between CD248-positive cells within ectopic lymphoid structures. However, the process of actively culturing non-inflamed control FLS may itself induce CD248 expression, with elevated CD248 being observed in the stromal cells of proliferating tissues. One final question we hoped to better address using FLS isolated by this method was whether they were representative of an intimal or subintimal FLS population. Intimal FLS can be distinguished from subintimal fibroblasts via a number of well-characterised markers. These include a high expression of the enzyme uridine diphosphoglucose dehydrogenase (UDPGD), and the surface markers VCAM-1 and cadherin-11 [46,47]. mRNA analysis of both cadherin-11 and VCAM1 confirmed that both of these markers were highly expressed in both the non-inflamed and inflamed K/BxN FLS cultures. Although these finding do not eliminate the possibility of a subintimal fibroblast contamination, they do suggest that our cultures are predominantly intimal. A more rigorous approach would be to stain these cells for cadherin-11 and UDPGD to assess their purity. Finally, as part of the characterisation of the FLS cultures, we were able to show that these cells were able to migrate through a matrigel membrane using a Boyden chamber method, using PDGF as a chemoattractant. This was further enhanced in the presence of TNF-α. The ability of FLS to invade into cartilage is a defining characteristic of inflamed synovial fibroblasts isolated from patients with rheumatoid arthritis [15]. PDGF and TGF-α are known to be potent stimulators of fibroblast migration and invasive behaviour [48]. Further experiments to characterise murine FLS might examine more closely their response to these secreted factors.

Using these well-characterised FLS cultures, we generated an inflammatory gene set focussing on IL-6, CCL-2, VCAM and ICAM-1. These cytokines, chemoattractants and adhesion molecules are considered to play a significant role in the pathology of RA and inflammatory disease [17-22,49].

In human RA and osteoarthritis (OA) FLS cultures the expression of these markers is elevated relative to fibroblasts isolated from non-inflamed tissues such as dermal and bone marrow fibroblasts [35]. Our FLS cultures mirrored these finding, with greater expression of inflammatory markers compared to fibroblasts isolated from non-inflamed synovium. This suggests that these cells are a useful model with relevance to human disease. Similarly, glucocorticoids were able to abrogate these elevated inflammatory markers, reducing their expression to a level that matched that of other mesenchymal cell lines [25].

TNF-α expression is highly upregulated in the RA synovium [25,26,35] and regulates proinflammatory cytokines such as IL-1, IL-6, IL-8 and granulocyte-macrophage colony-stimulating factor (GM-CSF) [50,51]. In particular, its importance has been demonstrated through the efficacy of TNF-α-depleting antibodies in the treatment of RA [52]. In this study, we examined a set of key inflammatory genes in murine FLS. In response to TNF-α all markers were significantly increased. The increase in IL-6 and CCl-2 was greater in FLS isolated from inflamed tissue relative to non-inflamed joints [35]. By contrast VCAM-1 and ICAM-1 demonstrated similar TNF-α induction in FLS isolated from inflamed and non-inflamed tissue. These data indicate that the persistent changes elicited on murine FLS by the inflammatory environment appear to be directed towards the systemic or local actions of IL-6 and CCL-2 mediated chemoattraction of monocytes populations. The idea of FLS derived IL-6 being of importance is of particular interest, as this cytokine has been shown to drive differentiation and activation of T helper (Th)17 cells in RA [21,22]. Consequently these data suggest there may be a similar mechanism involved in K/BxN arthritis. IL-6 secretion by FLS followed an identical pattern to changes in mRNA expression. These data highlight the use of IL-6 as an effective marker reflecting our results in the overall inflammatory gene set. Indeed, in multiple cell types including the uncommitted mesenchymal line C2C12, the partially differentiated osteoblast line MC3T3-E1 and control FLS isolated non-inflamed FLS, all had significantly lower levels of IL-6 compared to FLS isolated from inflamed joints. Certainly the K/BxN FLS possessed a greater capacity to respond to TNF-α confirming that these cells maintain an activated inflammatory phenotype compared to other mesenchymal cell lines.

As basal IL-6 expression was relatively high in K/BxN FLS, as well as being sensitive to both pro- and ant-inflammatory intervention, we concluded this cytokine may make a useful marker of inflammatory activation in murine FLS. Time course analysis and dose responses of IL-6 mRNA expression in response to corticosterone and TNF-α demonstrated changes with time and dose occurred across ranges that are considered to be physiological and/or that have been shown to be reached within inflamed tissues [53]. The findings within this study should facilitate the selection of appropriate doses and time points for readouts using murine FLS.

We also investigated the stability of the inflammatory phenotype in FLS with prolonged culture. Our data demonstrate significant macrophage contamination in primary cultures up to passage four. From this point all inflammatory genes examined remained stable through to passage seven. Beyond passage seven, several of the inflammatory genes began to show a significant decrease in expression. Consequently, based on these data, we would recommend use of murine FLS between passages four and eight. These finding are very similar to the subculture viability observed in human FLS.

In addition to the collagenase and *in vitro *culture method of FLS isolation used in this paper, several other methods have been successfully applied for the purpose of human synovial fibroblast culture. Of particular interest is the CD14-negative selection method where magnetic beads are used to remove synovial macrophages from the culture [34]. This can be further coupled with CD90.2-positive selection to yield an even purer FLS population. This has the advantage of removing many of the primary contaminating cell types early on in FLS culture, negating the need for prolonged subculture and *in vitro *expansion, potentially providing a cell population that is more representative of *in vivo *synovial fibroblasts. Future work in the K/BxN mice might utilise this methodology to compare and improve the characterisation of FLS isolated from inflamed synovium. The uses of synovial fragments for direct FLS culture outgrowth have also proved a viable method of isolation in human inflammatory disease. Unfortunately, this method is more complicated in murine inflammatory models due to the reduced quantities of inflamed synovium within murine joints.

The use of murine FLS have been instrumental in delineating the mechanisms of inflammatory bone loss. These include observations by Wei *et al. *and Li *et al. *demonstrating proinflammatory mediated signalling can induce RANKL secretion by FLS, increasing osteoclast activity [9,11]. More recently, seminal work by Diarra *et al. *identified proinflammatory NFκ-B signalling being important in increasing DKK-1 secretion by murine FLS, interfering with osteoblast maturation [10]. Many more studies have focused on the roles FLS play in pathophysiology of inflammatory disease exploring their contribution to leukocyte activation and driving the inflammatory process [54-56]. Importantly, work focusing on murine FLS culture in inflammatory models have provided greater insights into synoviocyte survival, migration, proliferation and contribution towards joint destruction that occur in human FLS-mediated disease [57-60].

By examining multiple proinflammatory genes in K/BxN FLS, we have created an inflammatory gene set that can be used to identify these cells *in vitro*. This information also provides a benchmark for examining how this profile is influenced by various pro- and anti-inflammatory interventions. This will help establish a normal standard profile from which to compare FLS isolated from transgenic mice. We examined FLS isolated from the K/BxN mice model of inflammation. This had the advantage of providing us with cells from tissues that have been exposed to prolonged inflammation, mirroring the situation in human inflammatory joint disease such as RA. Encouragingly the inflammatory gene expression observed in murine FLS were very similar to that seen in human FLS cultures, responding in an identical manner as previously reported for both TNF-α and the glucocorticoid corticosterone [25]. Additionally, these markers were maintained in the same way as with human culture over prolonged culture [25,34]. Consequently these data supports the use of these cells in ongoing studies looking at the mechanism of inflammation, where differences between murine and human can be common. It is currently unclear how the K/BxN FLS isolated here compare to FLS isolated from other inflammatory models such as collagen antibody-induced arthritis (CAIA) and antigen-induced arthritis (AIA). In human disease, FLS have been shown to possess different inflammatory profiles and responses between disease states such as OA and RA. Consequently, future studies might benefit from similar characterisation of FLS in a panel of inflammatory mouse models.

## Conclusions

Fibroblast-like synoviocytes have been shown to play a key role in the pathophysiology of inflammatory disease. In this study, we have characterised FLS isolated by collagenase digestion of synovial tissue from the K/BxN inflammatory mouse model. These were shown to respond to pro- and anti-inflammatory stimuli and express stromal and inflammatory markers in a manner that closely resembled that seen in human FLS culture. Consequently, this study provides characterisation and culture guidelines that support the growing body of work on murine FLS to model human inflammatory disease.

## Abbreviations

AIA: antigen-induced arthritis; BGLAP: bone gamma-carboxyglutamic acid-containing protein; BSA: bovine serum albumin; CAIA: antibody-induced arthritis; CCL-2: chemokine ligand 2; DMEM: Dulbecco's modified Eagle's medium; ELISA: enzyme-linked immunosorbent assay; FBS: fetal bovine serum; FLS: fibroblast-like synoviocytes; GM-CSF: granulocyte-macrophage colony-stimulating factor; ICAM-1: intercellular adhesion molecule 1; IgG: immunoglobulin G; IL: interleukin; PDGF: platelet-derived growth factor; OA: osteoarthritis; RA: rheumatoid arthritis; RANKL: receptor activator of nuclear factor kappa-B (NFκ-B) ligand; RT-PCR: reverse transcription polymerase chain reaction; Th: T helper; TNF-α: tumour necrosis factor alpha; UDPGD: uridine diphosphoglucose dehydrogenase; VCAM-1: vascular cell adhesion molecule 1; WT: wild-type.

## Competing interests

The authors declare that they have no competing interests.

## Authors' contributions

RSH derived initial primary cultures, cultured cells, extracted and analyzed RNA and co- wrote the manuscript. CH maintained cell culture and performed ELISA assays. YL extracted and analysed mRNA. SS performed genotyping on animals. JT managed animal handling and determined clinical scores. SG performed immunohistochemistry. CFY performed matrigel invasion assays. PMS, KR, MSC, MSJ and HZ developed the initial hypothesis. MJS, MSC and HZ co-wrote and analysed data. All authors read and approved the final manuscript.

## Supplementary Material

Additional file 1 **Unedited mRNA gel images**. Unedited gels for the mRNA expression of 18S, prolyl 4-hydroxylase (P4H), CD248, osteocalcin (OCN) and CD68 determined by standard RT-PCR at 35 cycles in one non-inflamed control FLS (wild-type), three K/BxN FLS lines (FLS 1 to 3), primary calvarial osteoblasts (OBs) and the macrophage cell line RAW 264.7 (MΦ). Click here for file

Additional file 2 **Positive control images for CD31 and CD68 staining**. Expression of CD31 (blue) in K/BxN fibroblast-like synoviocytes (FLS) culture determined by confocal immunofluorescence and in murine lung by immunohistocemistry, CD68 (red) in K/BxN FLS and in RAW CD68 +ve cells determined by confocal immunofluorescence. Nuclei are counterstained in green. Images shown are representative of three separate FLS cell lines. Click here for file

Additional file 3 **mRNA expression of CD68, cadherin 11 and IL-6**. **(a) **Fold change in mRNA expression of the macrophage surface marker CD68 in K/BxN fibroblast-like synoviocytes (FLS) at passages 3 and 4 determined by real-time RT-PCR. For each gene product data were normalized for levels of the housekeeping gene 18S rRNA and are presented as fold change in expression (± standard error) relative to the P2 control. Results shown are the combined duplicates of two K/BxN FLS. **(b) **mRNA ΔCt values for cadherin 11 in three separate wild-type non-inflamed and three K/BxN FLS cell lines after 16 hr treatments with either vehicle or TNF-α at 10 ng/ml. **(c) **Fold change in IL-6 mRNA expression over 0, 2, 4, 8, 16 and 24 hr following treatment with either corticosterone (100 nmol/l) or TNF-α (10 ng/ml). Results shown are the combined duplicates of three separate FLS lines. Data were normalized for levels of the housekeeping gene 18S rRNA and are presented as fold change in expression (± standard error) relative to the 0 hr control. *P < 0.05, **P < 0.001 versus respective untreated control. Click here for file
