# Supplementary figures and images for: Characterisation of fibroblast-like synoviocytes from a murine model of joint inflammation
Source: Arthritis Res Ther. 2013 Jan 29;15(1):R24. doi: 10.1186/ar4158 (PMC3672796; doi:10.1186/ar4158)

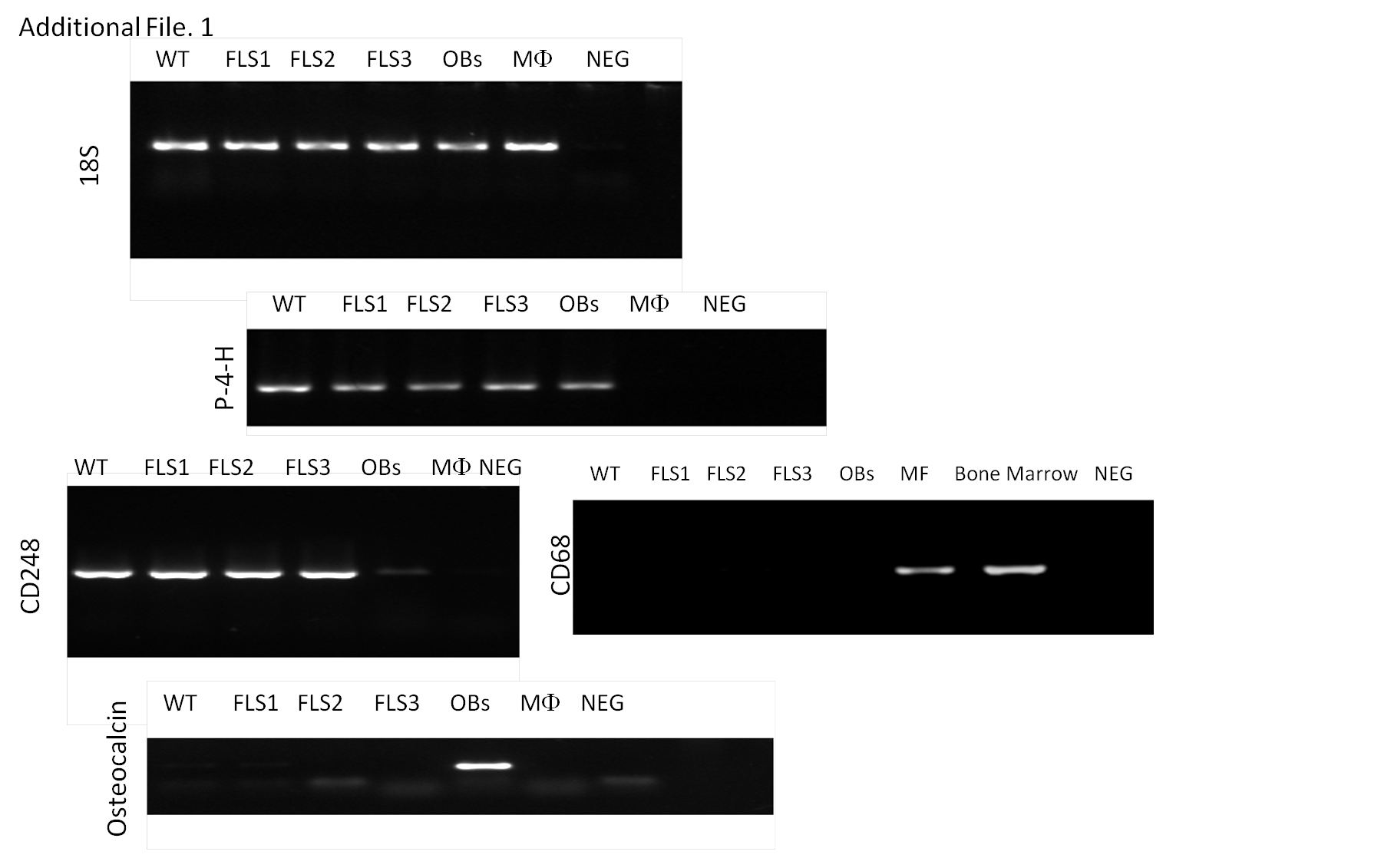

Supplement: Additional file 1 — Unedited mRNA gel images. Unedited gels for the mRNA expression of 18S, prolyl 4-hydroxylase (P4H), CD248, osteocalcin (OCN) and CD68 determined by standard RT-PCR at 35 cycles in one non-inflamed control FLS (wild-type), three K/BxN FLS lines (FLS 1 to 3), primary calvarial osteoblasts (OBs) and the macrophage cell line RAW 264.7 (MΦ). [file ar4158-S1.TIFF]

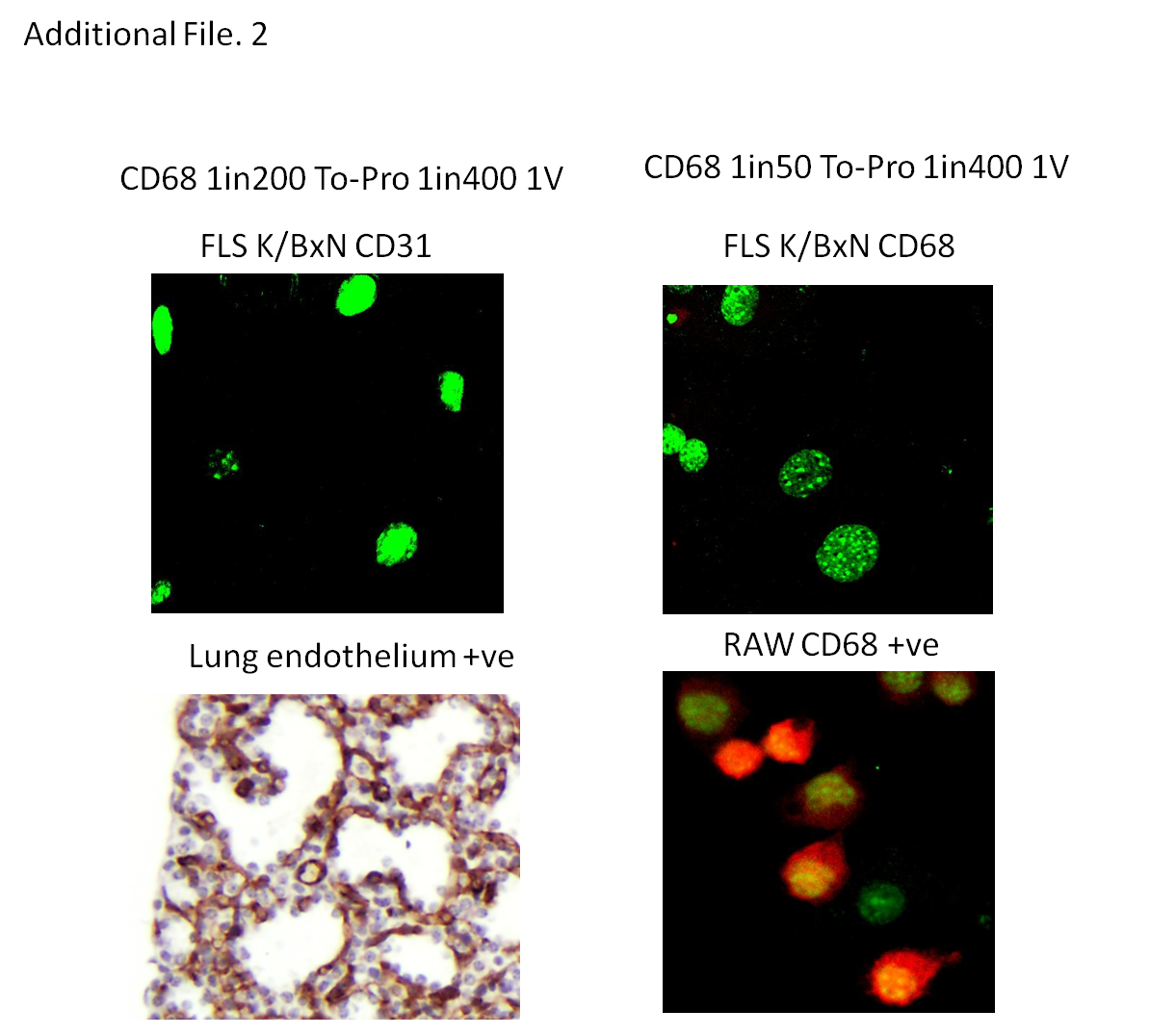

Supplement: Additional file 2 — Positive control images for CD31 and CD68 staining. Expression of CD31 (blue) in K/BxN fibroblast-like synoviocytes (FLS) culture determined by confocal immunofluorescence and in murine lung by immunohistocemistry, CD68 (red) in K/BxN FLS and in RAW CD68 +ve cells determined by confocal immunofluorescence. Nuclei are counterstained in green. Images shown are representative of three separate FLS cell lines. [file ar4158-S2.TIFF]

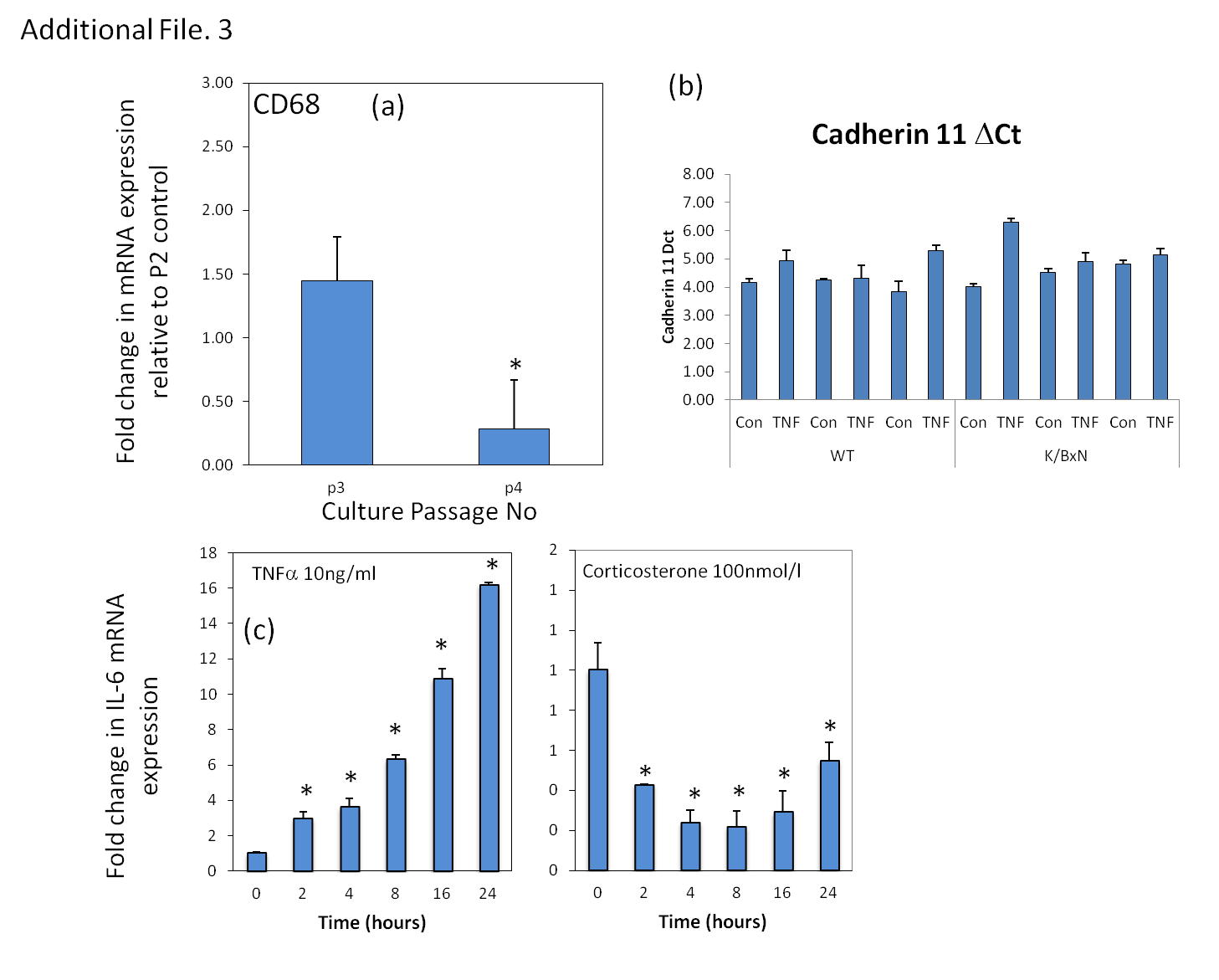

Supplement: Additional file 3 — mRNA expression of CD68, cadherin 11 and IL-6. (a) Fold change in mRNA expression of the macrophage surface marker CD68 in K/BxN fibroblast-like synoviocytes (FLS) at passages 3 and 4 determined by real-time RT-PCR. For each gene product data were normalized for levels of the housekeeping gene 18S rRNA and are presented as fold change in expression (± standard error) relative to the P2 control. Results shown are the combined duplicates of two K/BxN FLS. (b) mRNA ΔCt values for cadherin 11 in three separate wild-type non-inflamed and three K/BxN FLS cell lines after 16 hr treatments with either vehicle or TNF-α at 10 ng/ml. (c) Fold change in IL-6 mRNA expression over 0, 2, 4, 8, 16 and 24 hr following treatment with either corticosterone (100 nmol/l) or TNF-α (10 ng/ml). Results shown are the combined duplicates of three separate FLS lines. Data were normalized for levels of the housekeeping gene 18S rRNA and are presented as fold change in expression (± standard error) relative to the 0 hr control. *P < 0.05, **P < 0.001 versus respective untreated control. [file ar4158-S3.TIFF]
